# No particular genomic features underpin the dramatic economic consequences of 17^th^ century plague epidemics in Italy

**DOI:** 10.1016/j.isci.2021.102383

**Published:** 2021-03-31

**Authors:** Andaine Seguin-Orlando, Caroline Costedoat, Clio Der Sarkissian, Stéfan Tzortzis, Célia Kamel, Norbert Telmon, Love Dalén, Catherine Thèves, Michel Signoli, Ludovic Orlando

**Affiliations:** 1 Centre for Anthropobiology and Genomics of Toulouse CAGT, UMR 5288, CNRS, Université Toulouse III Paul Sabatier, Faculté de Médecine Purpan, Bâtiment A, 37 allées Jules Guesde, 31000 Toulouse, France; 2 Institute for Advanced Study in Toulouse IAST, Université Toulouse I Capitole, Esplanade de l’Université, 31080 Toulouse cedex 06, France; 3 Anthropologie bio-culturelle, droit, éthique et santé ADES, UMR 7268 CNRS EFS, Aix-Marseille Université, Faculté de Médecine, Secteur Nord Bâtiment A CS80011, Boulevard Pierre Dramard, 13344 Marseille Cedex 15, France; 4 Ministère de la Culture et de la Communication, Direction Régionale des Affaires Culturelles de PACA, Service Régional de l’Archéologie, 23 bd du Roi René, 13617 Aix-en-Provence cedex, France; 5 Centre for Palaeogenetics, Svante Arrhenius väg 20C, 10691 Stockholm, Sweden; 6 Department of Bioinformatics and Genetics, Swedish Museum of Natural History, Box 50007, 10405 Stockholm, Sweden

**Keywords:** Paleontology, Genomics, Microbiology

## Abstract

The 17^th^ century plague epidemic had a particularly strong demographic toll in Southern Europe, especially Italy, where it caused long-lasting economical damage. Whether this resulted from ineffective sanitation measures or more pathogenic *Yersinia pestis* strains remains unknown. DNA screening of 26 skeletons from the 1629-1630 plague cemetery of Lariey (French Alps) identified two teeth rich in plague genetic material. Further sequencing revealed two *Y. pestis* genomes phylogenetically closest to those from the 1636 outbreak of San Procolo a Naturno, Italy. They both belonged to a cluster extending from the Alps to Northern Germany that probably propagated during the Thirty Years war. Sequence variation did not support faster evolutionary rates in the Italian genomes and revealed only rare private non-synonymous mutations not affecting virulence genes. This, and the more heterogeneous spatial diffusion of the epidemic outside Italy, suggests environmental or social rather than biological causes for the severe Italian epidemic trajectory.

## Highlights

• DNA screening of 26 individuals from a 1629-1630 French Alps cemetery for plague
• Sequencing two *Yersinia pestis* plague genomes at 2.3- and 13.7-fold coverage
• These genomes are phylogenetically closest to those from the 1636 Italian outbreak
• No genetic changes explain the particularly increased spread of the disease in Italy

## Graphical abstract

**Figure:**
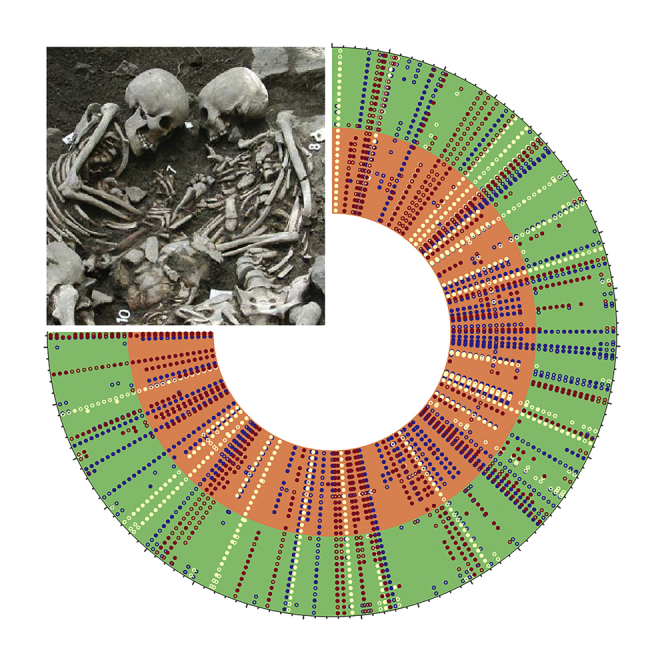

## Introduction

With the advent of next-generation DNA sequencing, ancient DNA research has moved from single locus studies to the characterization of the complete genomes of ancient individuals, including from extinct hominids such as Neanderthals and Denisovans (see Orlando et al., 2021 for a review). The variation present in the genome of ancient individuals now provides a novel type of historical source that can help rewrite the history of population movements across the Old World and into the New World (see Nielsen et al., 2017 for a review). Time-stamped genome data have also provided unprecedented resolution to the study of the process by which plants and animals have been domesticated, selected and propagated around the world (see Frantz et al., 2020 and Kistler et al., 2020 for reviews). As the DNA fragments of ancient pathogens can survive together with those from their hosts, ancient genomic data have largely contributed to better understand past epidemics. Such data have not only contributed to solve the mysterious origins of past epidemics, such as that, that have decimated Mexican populations following their first contact with Europeans in the 16^th^ century CE (Common Era) (Vågene et al., 2018) but also to track their evolutionary dynamics and the genetic changes that could facilitate transmission and may have affected virulence (see Spyrou et al., 2019a for a review).

Together with *Mycobacterium tuberculosis* (the agent of tuberculosis), *Yersinia pestis* (the agent of plague) represents the bacterial pathogen that thus far has received most attention in ancient DNA research. Although historians have identified the Justinian plague of the sixth century CE as marking the beginning of the first major plague pandemics, ancient DNA data have revealed that plague pathogens had in fact already started to infect human populations thousand years earlier, between the third and sixth millennium BCE (Before Common Era) (Rasmussen et al., 2015; Andrades Valtueña et al., 2017; Spyrou et al., 2018; Rascovan et al., 2019). Additionally, genome sequencing has revealed a pathogenic genetic toolkit much different then than at the time of the Justinian plague (Wagner et al., 2014; Feldman et al., 2016; Namouchi et al., 2018; Keller et al., 2019) and the infamous Black Death (Bos et al., 2011, 2016; Spyrou et al., 2016, 2019b; Guellil et al., 2020; Morozova et al., 2020; Susat et al., 2020), which marked the beginning of the so-called second pandemic by decimating 30-60% of the European population in the 14^th^ century CE (Eckert, 1978; Benedictow, 2004). For example, the absence of the *ymt* locus, which is normally present on the pMT1 plasmid and is key for the transmission of the disease by fleas (Sun et al., 2014), suggested different transmission modes for plague epidemics during the Bronze Age and the Iron Age (Rasmussen et al., 2015; Andrades Valtueña et al., 2017). Comparative genomic work has also provided examples of convergent evolution between strains from the first and second pandemics, including the parallel loss of magnesium transporters essential for survival into the macrophage phagosome (Keller et al., 2019; Spyrou et al., 2019b). Furthermore, strains from the 14^th^ century CE have been found remarkably homogeneous genetically across Europe and the Caucasus, suggesting a history of extremely rapid spread from a unique source (Bos et al., 2016). Genome characterization of additional strains accompanying the epidemic waves from the following centuries, and until the 18^th^ century CE (Biraben, 1975), have been found to form at least two main phylogenetic groups, potentially indicative of different origins from strains descending from the Black Death and either surviving in Europe (Bos et al., 2016) or in nearby foci (Guellil et al., 2020, see also Barbieri et al., 2020 for a review).

The second pandemic represents the plague pandemic that is currently best documented at the genetic level, with over 70 complete ancient plague genomes hitherto sequenced. Despite this, many areas require further research. One such area relates to the particular historical trajectory that plague epidemics from the 17^th^ century CE have had in Italy. There, the pathogen has been reported to have been more pervasive geographically than in most other European regions, especially in 1629-1631 CE where it caused a massive demographic impact in both the main cities (e.g. 62% mortality in Verona (Donazzolo and Saibante, 1926) and around 50% in Bologna, Mantua, Pavia (Del Panta and Livi Bacci, 1977) or Parma (Lucchetti et al., 1998)) and the countryside (Manfredini et al., 2002; Alfani and Murphy, 2017). This unleashed a long-lasting economic crisis, as tax incomes became considerably reduced and cities could not be rapidly repopulated from nearby villages so as to provide the manpower yet necessary to sustain their activities (Alfani, 2013). This particular context has been proposed to have marked the beginning of the Great Divergence between Italy and the other European countries, in which economies could restart much quicker and could, for some, benefit from established and growing colonial empires. As the Italian economy and institutions were amongst the best in the continent at the time when the epidemic struck (e.g. cities were equipped with permanent health boards from the 15^th^ century CE (Cipolla, 1976; 1981) and anti-plague tracts and other measures available at the time have been largely inspired from Italian publications (Cohn, 2009)), historians have proposed that the emergence of a new, more virulent strain may have contributed to the particularly dramatic impact measured in Italy (Alfani, 2013). In this study, we sequenced two complete plague genomes from individuals who died in the French Alps in 1629-1630 CE. These genomes were phylogenetically closest to those previously characterized in Italy in the following decade (Guellil et al., 2020). This provided us with a unique opportunity to investigate whether or not the pathogen developed a particularly harmful genetic set that could explain the dramatic epidemic striking Italy at the time.

## Results and discussion

### Genomic and metagenomic screening

**Figure 1:**
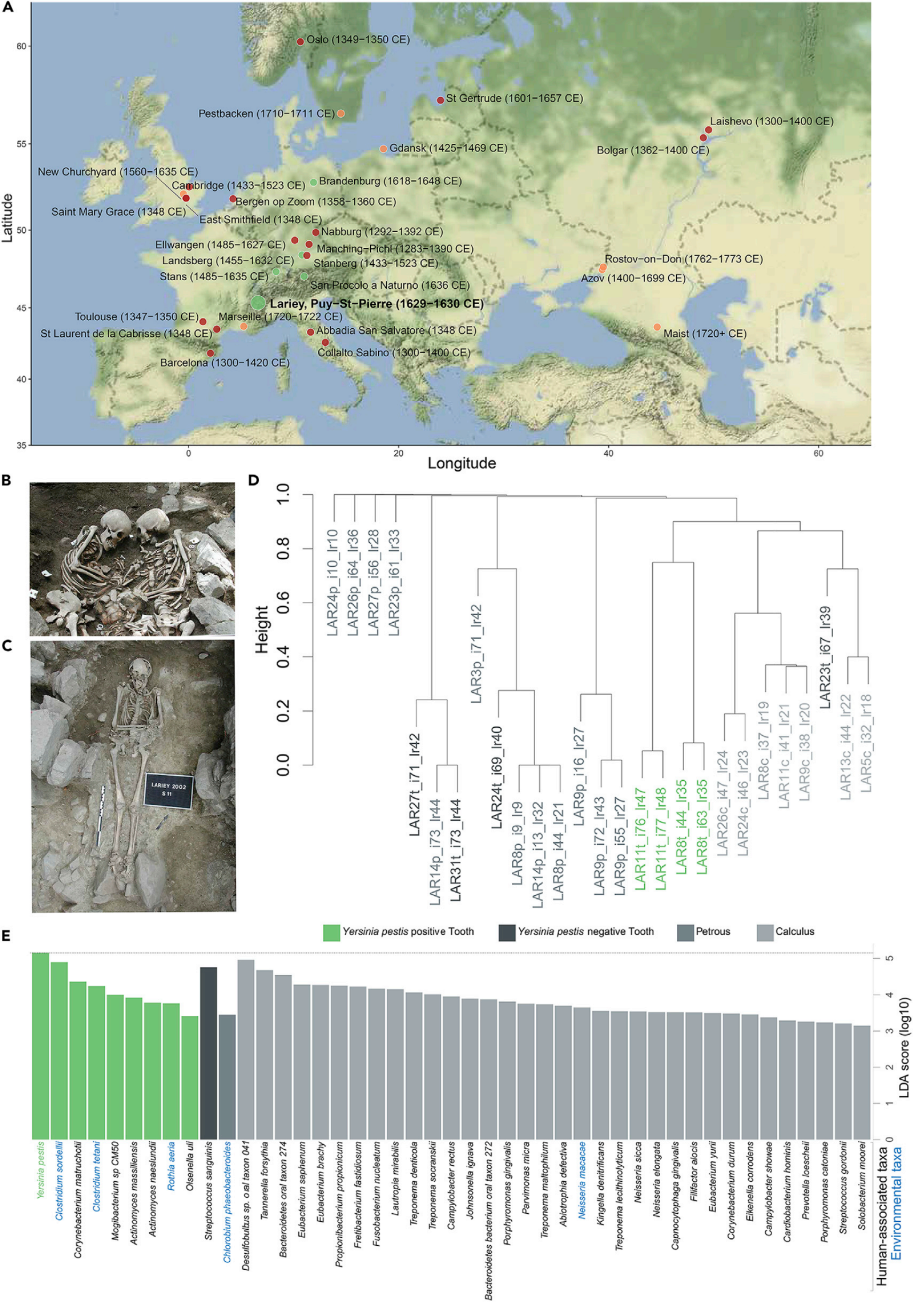
Sample information and metagenomic analyses (A) Map showing the location and temporal range of the Lariey-Puy-Saint-Pierre cemetery together with previously published second pandemic plague genomes. Colors are indicated with respect to Spyrou et al. (2019b) and according to the main phylogenetic clusters shown on Figure 3. (B) Multiple burial showing the LAR8 individual (right). (C) Simple burial showing the LAR11 individual. (D) Hierarchical clustering dendrogram of Bray-Curtis distances between MetaPhlAn2 (Truong et al., 2015) bacterial abundance profiles (10,000 bootstrap pseudo-replicates) and disregarding abundances below 1%. All clusters are supported with a pvclust (Suzuki and Shimodaira, 2006) approximately unbiased p value of 100. (E) LEfSe (Segata et al., 2011) Linear Discriminant Analysis indicating those microbial species with most contrasted abundance patterns (LDA scores >3). Higher log10-LDA scores identify those bacterial species contributing the most to the differences in the metagenomic content of teeth positive or negative for *Yersinia pestis*, petrosal and dental calculus remains. The source of each species was predicted on the basis of the literature and conservatively categorized as ‘*environmental’* whenever both sources were likely. See also Figures S1 and S2.

A total of 12 ancient human petrosal bones, 10 teeth and 7 dental calculus from 15 individuals excavated at the Lariey-Puy-Saint-Pierre cemetery (France) were prepared in ancient DNA facilities for shallow shotgun sequencing on the MiniSeq Illumina instrument (transparent methods; Table S1 and Figure S1). This cemetery represents the only French site from the 17^th^ century CE that can unambiguously be linked to the 1629-1630 CE plague pandemic (Signoli et al., 2003a) (Figures 1A–1C). Sequence mapping against the hs37d5 human reference genome revealed substantial variation in human DNA content, with petrosal bones showing maximal proportions (median = 72.43%, range = 1.57%–97.96%), followed by teeth (median = 2.67%, range = 0.02%–18.82%) and dental calculus (median = 0.08%, range = 0.05%–0.34%; Table S1A). This is in line with the generally better postmortem DNA preservation reported for petrosal bones (Pinhasi et al., 2015) and dental calculus deriving mainly from oral bacterial biofilms (Warinner et al., 2015). The high variation in the human endogenous DNA content measured across samples of similar types supports that DNA preservation is driven by micro-environmental factors instead of global physico-chemical parameters characteristics of the site.

Bacterial taxonomic profiling with metaBIT (Louvel et al., 2016) against the MetaPhLAn2 database (Truong et al., 2015) indicated marked differences in 29 samples successfully characterized, with only one single tooth from individual LAR23 and one dental calculus tissue from individual LAR13 clustering together with modern dental plaque samples (Figure S2). Most of the other remains occupied a central position in the Principal Coordinate Analysis, indicating low diversity probably due to the presence of environmental microbes contaminating archaeological remains after death. However, abundance profiles possibly suggested non-negligible DNA proportions of *Yersinia pestis* in the teeth of two individuals LAR8 (10.8–10.9%) and LAR11 (40.7–42.1%). Polymerase chain reaction (PCR) amplifications of a 133-bp fragment located in the pPCP1 *pla* gene returned positive results on the tooth DNA extracts of these two individuals. They remained, however, negative across all the other tooth extracts tested, except one (LAR27), including 13 additional extracts that were not previously screened by sequencing (Table S1). The fact that only a small fraction of the individuals was detected positive for plague reflects the extensive postmortem DNA fragmentation and the relatively limited power of shotgun sequencing in identifying plague from DNA extracts dominated by human and/or environmental bacterial templates.

The microbial profiles from the LAR8 and LAR11 teeth were closer to each other than to any other material sequenced, including other teeth (Figure 1D). *Yersinia pestis* was furthermore confirmed through Linear Discriminant Analyses in LEfSe (Segata et al., 2011) as the top bacterial species driving the abundance profiles of those two teeth versus those that were negative for *Yersinia pestis* and the other remains. The presence of a number of oral microbes, such as *Tannerella forsythia* and *Treponema denticola*, was also characteristic of microbial profiles obtained from ancient dental calculus tissues (Figure 1E).

Read mapping against the CO92 plague reference genome with the stringent parameters described in previous studies (Spyrou et al., 2019b; Keller et al., 2019) revealed clear signatures of postmortem DNA damage, confirming the likely presence of the pathogen in both the LAR8 and LAR11 individuals (Figure S3). Such signatures appeared mainly in the form of an excess of cytosines at those genomic positions preceding mapped reads and increased (although faint) C-to-T (G-to-A) nucleotide mis-incorporation rates at read starts (ends) (Briggs et al., 2007). This is so because DNA extracts were treated with the USER enzymatic mix that breaks the DNA backbone downstream of those cytosine residues that have been deaminated after death (Rohland et al., 2015). Interestingly, mapDamage (Jónsson et al., 2013) inferred higher nucleotide mis-incorporation rates in human sequence alignments than in plague data. This was not indicative of different postmortem DNA degradation in the host and the plague genome. Instead, this reflected faster postmortem cytosine deamination at methylated CpG dinucleotides (Smith et al., 2014; Seguin-Orlando et al., 2015), which are found in the human but not the plague genome (Figure S4).

Altogether, our results suggested the likely presence of *Yersinia pestis* DNA in the tooth extracts of the LAR8 and LAR11 individuals. The virtual absence of plague DNA in the petrosal bones of individuals otherwise positive for the infection confirms previous reports on plague (Margaryan et al., 2018). The absence of plague DNA in the dental calculus of individuals otherwise positive for the infection contrasts, however, with the recent successful characterization of *Mycobacterium leprae* from ancient dental calculus remains (Fotakis et al., 2020). This likely reflects the different etiology of the diseases, with leprosy, but not plague, causing lesions in the mucous membranes of the upper respiratory tract (de Abreu et al., 2006).

### Plague genome sequencing

**Figure 2:**
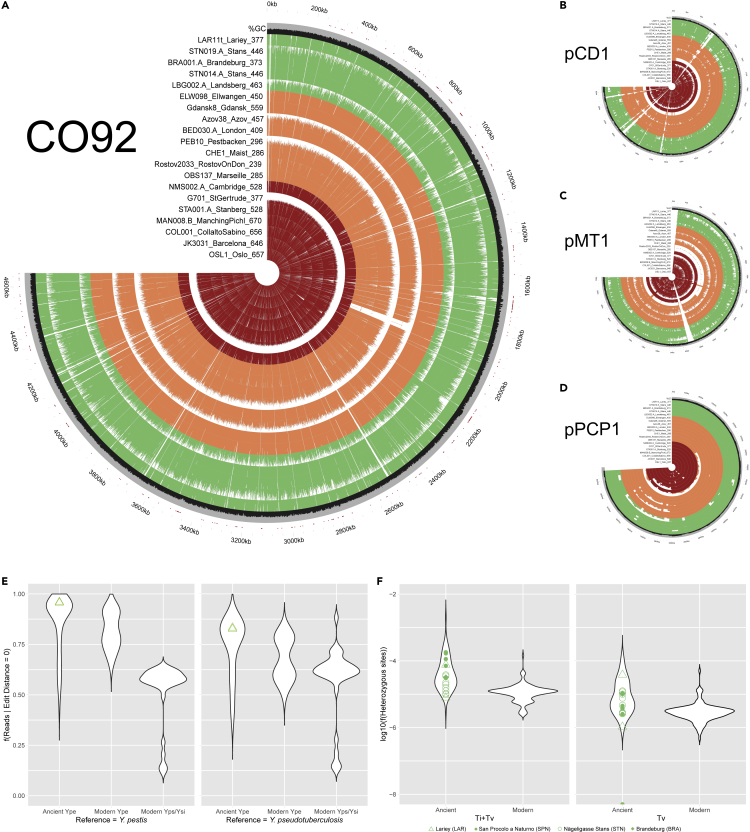
Plague chromosome and plasmid sequence coverage (A) Coverage and %GC variation along 1,000 bp windows along the CO92 plague reference genome ( GenBank: NC_003143.1) (Parkhill et al., 2001). Average depth-of-coverage was calculated using Paleomix (Schubert et al., 2014). %GC composition was calculated using seqtk (https://github.com/lh3/seqtk). Circular plots were traced using the circlize library in R (Gu et al., 2014) (max = 10-fold). In case multiple genomes are available for one site, the genome showing maximal coverage is shown. (B) Coverage and %GC variation along 100 bp windows along the pCD1 plague plasmid (GenBank: NC_003131.1). (C) Same as panel B, except that the pMT1 plasmid is shown (GenBank: NC_003134.1). (D) Same as panel B, except that the pPCP1 plasmid is shown (GenBank: AL109969.1). (E) Edit distance profiles obtained when mapping the sequence data underlying the LAR8 and LAR11 genomes and a comparative panel of 78 ancient and 155 modern *Yersiniapestis* (Ype), *Yersinia pseudotuberculosis* (Yps) and *Yersinia similis* (Ysi) sequence datasets aligned against the Ype and Yps reference genomes, respectively (Table S2). Edit distance distributions were considered as long as based on a minimum of 500 reads. (F) Heterozygosity profiles of the LAR8 and LAR11 genomes and a comparative panel of 78 ancient and 129 modern *Yersinia pestis* sequence datasets aligned against the CO92 *Yersinia pestis* reference chromosome (Table S2). Transition SNPs were disregarded to account for the differential rates of postmortem DNA damage amongst the comparative panel.

The fraction of plague DNA sequences identified during our preliminary screening indicated that complete bacterial genomes could be obtained with reasonable sequencing efforts. Further stringent mapping of an additional 139.0 and 188.6 million reads generated on the NovaSeq instrument from the DNA content of LAR8 and LAR11 tooth libraries resulted in the characterization of two plague genomes at an average 2.3-fold and 13.7-fold coverage, respectively (Figures 2A–2D). The pCD1, pMT1 and pPCP1 plasmids were also sequenced at 4.7–219.3-fold coverage in both individuals (Table S2). Edit distance distributions confirmed the genetic proximity to *Yersinia pestis* relative to close outgroups, such as *Yersinia pseudotuberculosis* and *Yersinia similis* (Figure 2E). Additionally, the fraction of heterozygous calls was limited in both individuals and comparable to that observed in modern individuals infected by a single bacterial strain and most ancient genomes previously characterized (Figure 2F). This indicated that a single infection rather than multiple co-infections from highly divergent strains likely caused the death of the two individuals.

### Human genome analyses

Further mapping of the tooth sequence data provided limited coverage of the human genome for the LAR8 and LAR11 individuals (0.377-fold and 0.085-fold, respectively). This was, however, sufficient to confirm previous sex determination of LAR8 as a female individual based on the shape of the coxal bone (Signoli et al., 2003b) and to identify LAR11, an immature individual who could not be sexed anatomically (Figure S1B), as a male individual. Both the presence of typical postmortem DNA damage profiles (Figure S3B) and the calculation of negligible contamination rates (≤1%) supported data authenticity. The latter were obtained on the basis of mitochondrial sequence variation for both individuals, or the X chromosome for the male individual (Tables S3 and S4). We found that the two individuals were not first- or second-degree relatives (Table S5) and projected onto the genomic variation of modern western European (Figure S5A), close to present-day French, Spanish, and Italian individuals. f3-Outgroup statistics also supported genetic affinities to present-day western Europeans (Figures S5B and S5C), and the two individuals carried mitochondrial (T1a1 and H2a1 for LAR8 and LAR11, respectively) and Y-chromosomal (R1b1a1b1 for LAR11) haplogroups that are relatively common in this region today (Tables S3 and S4).

### Phylogenetic analyses

**Figure 3:**
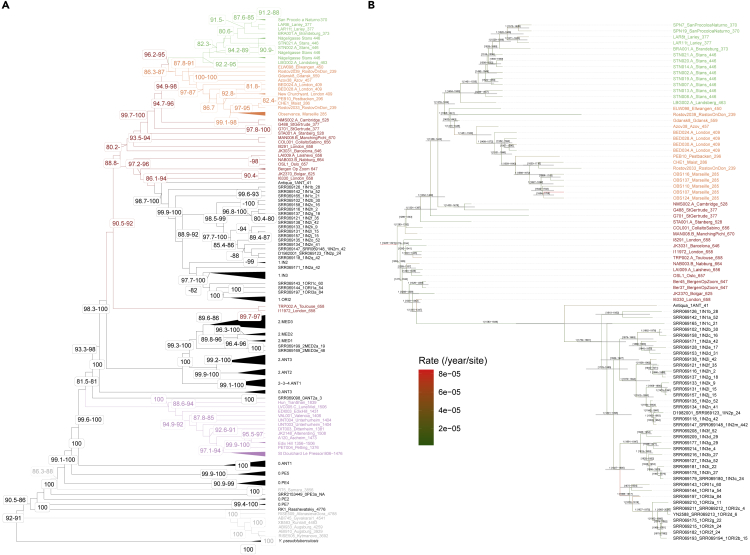
Phylogenetic reconstructions (A) Maximum Likelihood topology returned by IQTree (Minh et al., 2020) using a TVM + F + R6 substitution model and a total of 21,279 polymorphic sites. Node supports are indicated using SH-aLRT support (Guindon et al., 2010) (left), as well as the ultrafast bootstrap approximation (Hoang et al., 2018) (right) when superior or equal to 80%. Only one value is shown when both supports are equal to 100%. (B) Consensus maximal clade credibility phylogeny obtained with BEAST v2 (Bouckaert et al., 2019) restricting the sequence alignment to ancient and modern plague genomes phylogenetic clustering with or descending from second pandemic strains.

We next placed the LAR8 and LAR11 plague genomes into the phylogeny of modern and ancient plagues using Maximum Likelihood reconstruction with IQTree (Minh et al., 2020) (Figure 3A). This confirmed previous findings showing that Neolithic-Bronze Age strains (Rasmussen et al., 2015; Andrades Valtueña et al., 2017; Spyrou et al., 2019a; Rascovan et al., 2019), and those strains underlying the first (Wagner et al., 2014; Namouchi et al., 2018; Keller et al., 2019) and second pandemics (Bos et al., 2011, 2016; Morozova et al., 2020; Spyrou et al., 2016, 2019b; Susat et al., 2020) had various evolutionary origins. Additionally, no phylogenetic structure was found amongst 14^th^ century CE second-pandemic strains. This is in line with their rapid, almost clonal spread across Europe at the time of the Black Death (Bos et al., 2016).

Post-Black-Death strains appeared, however, differentiated into two main phylogenetic groups. The LAR8 and LAR11 plague genomes were nested within a first phylogenetic cluster grouping together strains retrieved from individuals buried during the late 15^th^ to the mid-17^th^ century CE in Italy (San Procolo a Naturno, SPN; 1636 CE), Switzerland (Stans, 1485-1635 CE), and Germany (Landsberg, 1455-1632 CE and Brandenburg, 1618-1648 CE) (Figures 1A and 3A). The second phylogenetic cluster included strains stretched throughout the Caucasus and Europe and spanning the mid-15^th^ to the late 18^th^ century CE. Bayesian phylogenetic analyses in BEAST v2 (Bouckaert et al., 2019) indicated that both clusters split between 1379 and 1434 CE (median = 1407 CE) while the most common recent ancestor of second pandemic strains most likely lived between 1228 and 1321 CE (median = 1283 CE) (Figure 3B; Table S2). We noticed that each of the second-pandemic and post-second pandemic clusters showed a clear temporal structure in which older genomes generally branched first. Interestingly, these data are in line with historical evidence indicating that plague would have circulated from Germany to France and Italy following Thirty Years War troops movements. This, and the coexistence of two differentiated phylogenetic clusters within Europe, suggest a history of outbreaks deriving from two different bacterial strains, both descending from the Black Death. The geographic restriction of the first phylogenetic cluster along the Alps and Germany (Figure 1A) may indicate the persistence of local foci in the region, possibly adapted to new rodent secondary hosts, as previously suggested (Carmichael 2014; Bos et al., 2016; Susat et al., 2020). It may, however, also reflect the insufficient sampling currently available throughout Europe, the Caucasus and Russia. Therefore, further work is required to test the possibility of alternative sources.

Interestingly, the two genomes from Lariey were monophyletic and appeared phylogenetically extremely close, suggesting that both individuals died during a single outbreak (Figure 3B). This contrasts to the situation reported in another site from the 17^th^ century CE of Latvia, where two local genetically divergent strains could be documented (G488 and G701) (Susat et al., 2020). Importantly, the LAR8 and LAR11 genomes were closest to those from SPN, characterized from individuals who died at this Italian location in 1636 CE. Both Lariey and SPN genomes seem directly related to one genome from Brandenburg (BRA001), Germany, that was sequenced from the remains of one foreign Swedish soldier who occupied the city in 1631 CE during the Thirty Years War (Spyrou et al., 2019b). The direct genetic connection found between these different genomes adds to multiple historical sources highlighting the role that this war played in spreading the disease (Wilson, 2009).

### Genome evolution in Italy

The sister phylogenetic relationship found between the SPN and Lariey genomes provided a unique opportunity to test the possibility of local biological adaptation for the plague circulating at the time in Italy, where the epidemic had a more profound demographic impact than in most other European countries (Alfani, 2013). There, the epidemic spread and killed in cities, hamlets and villages alike, which considerably limited the repopulation potential and the available workforce of the country. This has been proposed to have significantly delayed the economic recovery of major city centers and to have played an important role in the economic divergence that followed between Italy and neighbor countries, especially those with increasing colonial power (Alfani, 2013; Parker, 2017).

**Figure 4:**
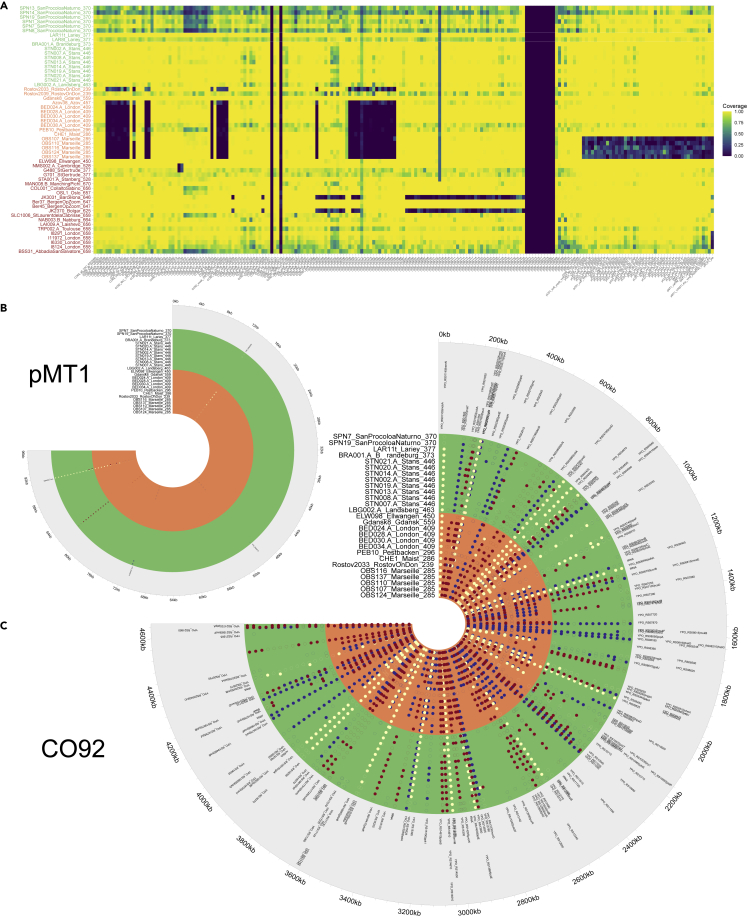
Genome composition of SPN, Lariey and other second pandemic plague genomes (A) Sequence coverage at 207 gene loci involved in the pathogen virulence and transmission. Coverage represents the fraction of the gene CDS at least covered once (0 = not covered, 1 = fully covered). (B) Non-synonymous (blue), synonymous (yellow) and intergenic (red) mutations present in a subset of 27 second pandemic ancient plague pMT1 plasmids. Open circles indicate sites not covered by at least 2 independent sequencing reads in an individual plasmid. The sequence data was trimmed and individual base quality scores rescaled in order to limit the potential impact of postmortem DNA damage. (C) Same as Panel B, except that those mutations affecting the CO92 reference chromosome are shown. See also Figure S6.

In order to assess potential differences in the gene composition of the SPN and Lariey plague genomes, we looked at patterns of coverage variation at 207 virulence loci (Figure 4A) (Cui et al., 2013). This approach confirmed the previously described deletion of the *mgt* and *mgtC* genes in several second pandemic strains (Spyrou et al., 2019b; Guellil et al., 2020). These deletions were, however, not present in the phylogenetic cluster that included the SPN and Lariey genomes. Some virulence factors showed limited coverage across several SPN genomes (irp1-irp8; Figure 4A). Since this was only the case for those genomes characterized at minimal average coverage (SNP1, SNP8, SNP13, and SPN14; 1.2–2.6-fold average coverage), it was indicative of local coverage drop due to limited sequencing efforts, rather than deletions. Therefore, the SPN strain likely did not benefit from the increased intracellular survival potential within macrophages associated with *mgtB* and *mgtC* loss (Ford et al., 2014). Likewise, the SPN genomes were not different from other genomes in their phylogenetic clusters, as those did not show any particular deletion of the *inv* gene either (Figure 4A). The product of this gene is involved in epithelial colonization in *Yersinia pseudotuberculosis* but not in *Yersinia pestis* (Simonet et al., 1996), and deleted strains were previously reported in post-Black Death strains from Cambridge, UK (Spyrou et al., 2019b) and St Gertrude, Latvia (Susat et al., 2020). Furthermore, it has been hypothesized that the coexistence of *pla*-deleted and regular pPCP1 plasmids in some post-Black Death plague strains may have reduced the rapid spread of the disease through flea vectors and favored the emergence of septicemic rather than bubonic symptoms (Susat et al., 2020). We recovered those coverage drops previously reported for some second pandemic strains and confirmed their presence in both the SPN and Lariey genomes, which all showed sequencing read spanning the whole locus (Figure S6). This suggests no *pla*-dependent differential transmission rates between those strains.

We finally established the list of private mutations found amongst SPN genomes compared to other second pandemic plague genomes, including from Lariey (Figures 4B and 4C, Table S6). This was restricted to the SPN7 and SPN19 genomes, as the only one sequenced at sufficient coverage to confidently identify SNPs (i.e. approximately ∼6.1-fold following trimming and base rescaling for handling possible nucleotide mis-incorporation arising from postmortem DNA damage; Table S2). Similarly, LAR8 was dismissed due to limited coverage. Only two non-synonymous mutations were common to the SPN7 and SPN19 genomes and not found in LAR11 or any other second pandemic genomes present in our comparative panel (Table S6). The first such mutation was located at the *treC* locus (CO92, NC_003143.1:4,130,262 G > A) (Figure 4C), a gene encoding the trehalose-6-phosphate hydrolase (also known as the alpha,alpha-phosphotrehalase). This enzyme is not known to affect virulence but is involved in starch and sucrose metabolism and acts as osmoprotectant in *Escherichia coli* (Rimmele and Boos, 1994). The second non-synonymous variant found specifically in SPN genomes only affected the YPO_RS00910 gene (pMT1, NC_003134.1:71,016A > G) (Figure 4B), which is involved in the type II toxin-antitoxin system RelE/ParE family toxin, a system ensuring stable plasmid inheritance for the bacteria (Guglielmini and Van Melderen, 2011). The SPN and Lariey genomes differed for a third mutation affecting one tRNA gene (CO92, NC_003143.1:3,336,035). This site was, however, found polymorphic across the other second pandemic strains and was, thus, an unlikely candidate for driving SPN-specific virulence phenotypes.

The relatively limited number of variants distinguishing the SPN genomes from those of Lariey and other second pandemic strains suggested no excessive accumulation of beneficial mutations along the SPN lineage. As hypermutability can, however, lead to the quicker emergence of beneficial mutations providing a fitness advantage to the pathogen in the co-evolutionary arms race against their host (Elena and Lenski, 2003), we further explicitly tested whether the SPN lineage displayed particularly accelerated evolutionary rates. Root-to-tip regression in TempEst (Rambaut et al., 2016) indicated temporality in the sequence data available for second pandemic strains (Supplemental information). However, BEAST analyses did not support any specific substantial shift in the mutational clock along the branch leading to the SPN cluster (Figure 3B). Our approach did not show limited sensitivity since a major acceleration in other phylogenetic branches could be detected. This, and the absence of private mutations affecting genes involved in DNA repair, rules out potentially beneficial hyper-mutator phenotypes amongst SPN strains. Hypermutability can thus be dismissed from the list of possible drivers of the increased damage to the Italian human population observed at the time.

### Conclusion

Overall, the Italian plague genomes from the 17^th^ century CE analyzed in this study showed only minute genetic differences with their closest evolutionary relatives. While the existence of other diverging strains taking over SPN cannot formally be ruled out without extensive genome sampling in Italy at the time, the relative genetic proximity amongst other second pandemic plague genomes suggests this as an unlikely alternative. The reason why more virulent strains would then remain endemic to Italy at the time of trans-European wars is also unclear. Overall, this suggests that the strains circulating in Italy during the Thirty Years War were likely not more virulent than their close phylogenetic relatives found in France, Switzerland and Germany. It follows that the underlying cause of the specific epidemic trajectory in Italy may not lie in the pathogen biology itself but rather in factors such as environmental, social and political that ultimately facilitated territorial pervasiveness and spread of the disease to villages, hamlets and cities altogether (Alfani, 2013). Previous work suggested that this extreme situation prevented the country from a quick demographic recovery, which limited the total production and fiscal income and resulted in important, long-lasting economical damage. Further work will be required to investigate the role of pathogens other than plague (e.g. typhus) and climate change in this crisis as the two other common scourges at the time.

### Limitations of the study

In this study, the sequence variation present among the ancient plague strains was only investigated following read alignment against one single reference genome and not through *de novo* genome assembly. Structural variants and their possible functional consequences, thus, remain overlooked. Additionally, the genetic diversity present in Italy during the 17^th^ century CE may not have been fully characterized from the sequence variation present in the single archaeological site of San Procolo a Naturno.

### Resource availability

#### Lead contact

Further information and requests for resources, material and reagents should be addressed and will be fulfilled by the lead contact, Ludovic Orlando (ludovic.orlando@univ-tlse3.fr).

#### Materials availability

This study did not yield new unique reagents.

#### Data and code availability

Raw sequence data and alignments are publicly available at the European Nucleotide Archive (ENA) under accession number ENA: PRJEB43291. Previously published genomic data used in this study are available at the sources referenced in the transparent methods. The software and computational procedures are detailed in the transparent methods, including the individual versions used as well as the command parameters.

## Methods

All methods can be found in the accompanying Transparent methods supplemental file.

## Supplemental information

Document S1. Transparent methods and Figures S1–S6

Table S1. Sample overview, including PCR screening and sequencing statistics A total of 26 ancient individuals were processed for ancient DNA analyses. Three main tissue types were analyzed, including dental calculus, petrosal bones and teeth, Ancient DNA extracts were treated with the USER enzymes so as to limit the impact of postmortem DNA damage on downstream sequence analyses. pla PCR indicates those extracts that were screened for the presence of a 133-bp long fragment within the pla gene, located on the pPCP1 plasmid (NA = Not Attempted; 0 = negative; 1 = positive). A total number of 45 blunt-ended dual indexed DNA libraries were prepared for sequencing (i = external P7 index; lr = pair of internal indices). Endogenous DNA contents (i,e, including both mitochondrial and nuclear genome reads) are provided following PCR duplicate removal and restricting counts to those high-quality alignments. Clonality refers to the fraction of the read alignments identified as PCR duplicates. $Non-collapsed read pairs count only for one single read, instead of two. ∗Minimum depth filter of 3 for haplogrep haplotype calling (instead of 5). The posterior mtDNA haplotype assignment probability is provided between parentheses, Related to Figure 1.

Table S2. Plague genome resources considered in the various sequence analyses Coverage estimates are provided following removal of PCR duplicates and filtering for high-quality DNA reads. Coverage estimates are also provided following read trimming and base quality score rescaling aimed at mitigating the presence of postmortem DNA damage in the sequence data. Genomes that are characterized with an average depth-of-coverage inferior to that of LAR8t are highlighted in gray. They were excluded from a series of analyses that require deeper sequencing. NA = Not Available, Related to Figure 2.

Table S3. Mitochondrial haplotypes and mitochondrial contamination estimates Contamination estimates are based on the methodology implemented in Schmutzi (Renaud et al., 2015). Mitochondrial haplotypes were inferred using haplogrep (Kloss-Brandstätter et al., 2011), Related to Figure 1.

Table S4. Y chromosome haplotype and nuclear contamination estimate Y chromosome haplotypes were inferred using Yleaf (Ralf et al., 2018). Contamination estimates are based on diversity measurements on the X chromosome, following the methodology presented by Rasmussen and colleagues (2011), Related to Figure 1.

Table S5. Relatedness between individuals as estimated in lcMLkin (Lipatov et al., 2015), Related to Figure 1

Table S6. Sequence differences amongst second plague pandemic genomes 0: Reference (REF) allele. 1: Single alternate (SNP) allele. 2, 3: Multiple non-reference alleles: Uncovered position. NS: Non-synonymous polymorphism. Syn: Synonymous substitution. ProPosition: Position of the amino acid. (AA) change within the protein primary sequence, Related to Figure 4.

## Footnotes

Supplemental information can be found online at https://doi.org/10.1016/j.isci.2021.102383.
