# Supplementary material for: No particular genomic features underpin the dramatic economic consequences of 17th century plague epidemics in Italy
Source: iScience. 2021 Mar 31;24(4):102383. doi: 10.1016/j.isci.2021.102383 (PMC8082092; doi:10.1016/j.isci.2021.102383)
Supplement: Document S1. Transparent methods and Figures S1–S6 [file mmc1.pdf]

iScience, Volume *24*

Supplemental information

No particular genomic features underpin the dramatic economic consequences of 17th century plague epidemics in Italy

Andaine Seguin-Orlando, Caroline Costedoat, Clio Der Sarkissian, Stéfan Tzortzis, Célia Kamel, Norbert Telmon, Love Dalén, Catherine Thèves, Michel Signoli, and Ludovic Orlando

Supplemental Figures

Figure S1. Preservation states of the cranial and post-cranial skeletons of the LAR8 and LAR11 individuals. (A) Sample LAR8. (B) Sample LAR11. Related to Figure 1.

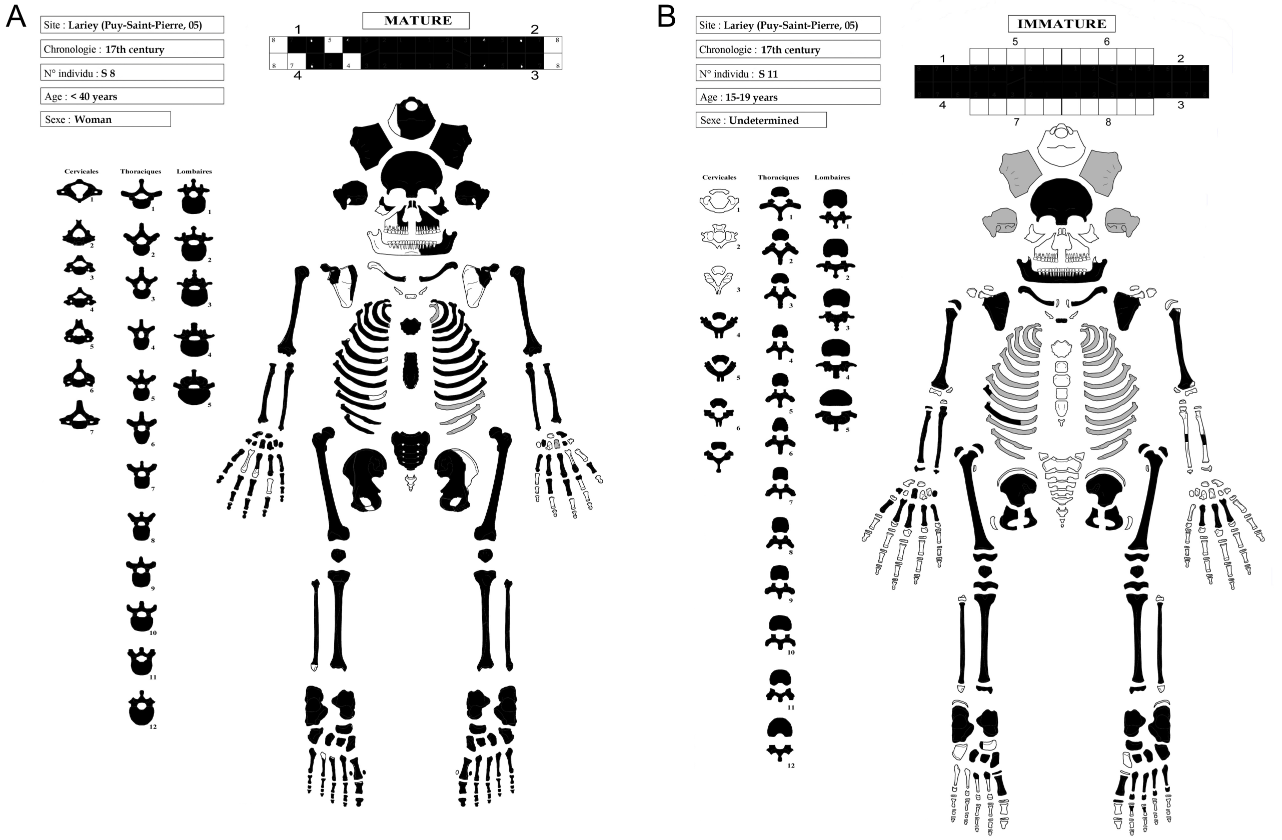

Figure S2. Genus-level Principal Coordinates Analysis (PCoA) of MetaPhlAn2 (Truong et al., 2015) bacterial abundance profiles. The 29 profiles obtained in this study are compared to a panel of 15 soil and 689 human-associated modern microbiota obtained from (Fierer et al., 2012; The Human Microbiome Project Consortium, 2012). Those remains analyzed here with shotgun sequencing DNA data are indicated with respect to the tissue originally sampled (c: dental calculus; p: petrosal bone, and; t: tooth) and the external (i) and internal indices (lr) used for constructing triple-indexed double-stranded DNA libraries. Related to Figure 1.

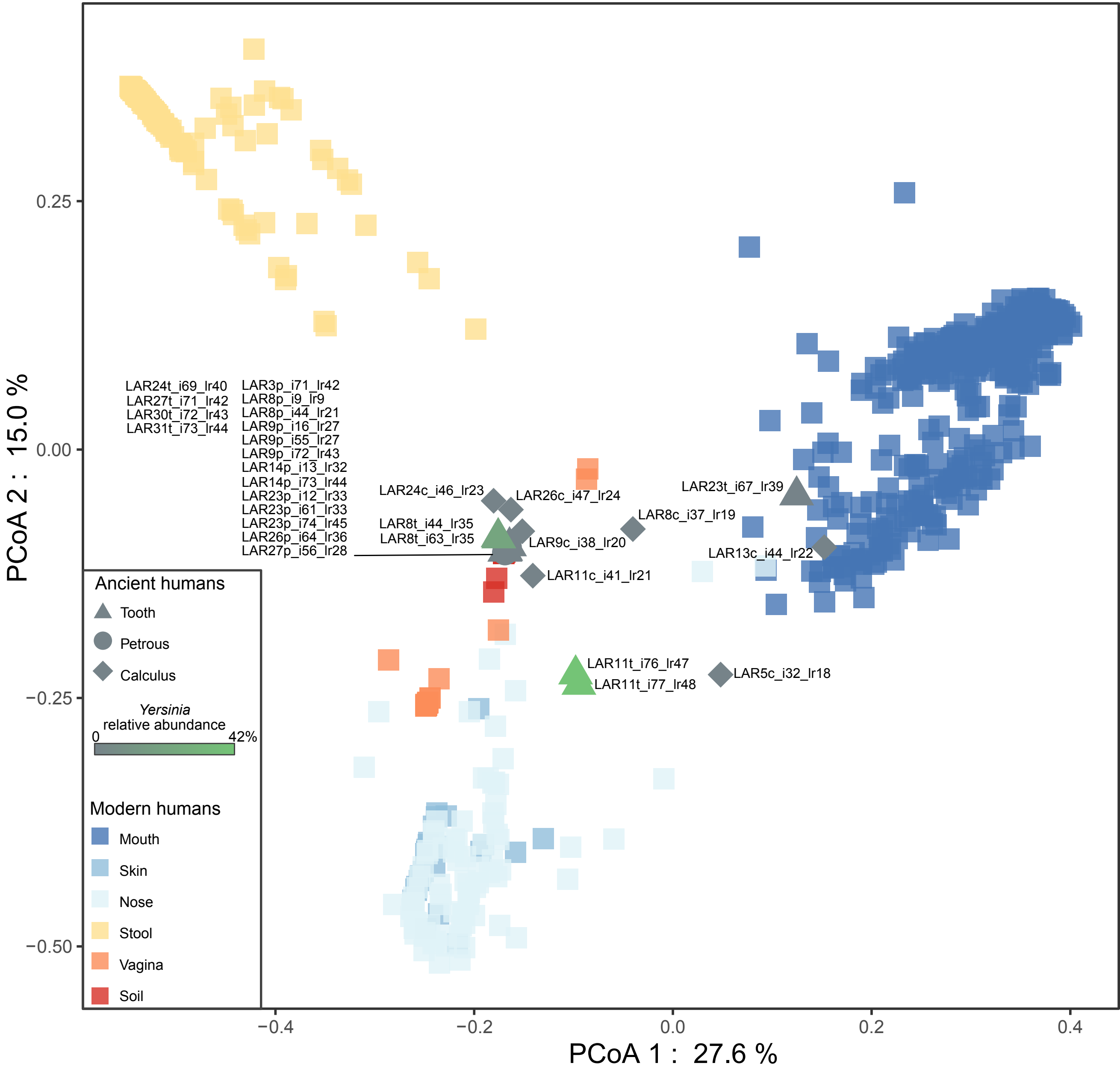

PCoA 2 : 15.0 %
PCoA 1 : 27.6 %
0.25
0.00
−0.25
−0.50
−0.4
−0.2
0.0
0.2
0.4
LAR24t_i69_lr40
LAR27t_i71_lr42
LAR30t_i72_lr43
LAR31t_i73_lr44
LAR3p_i71_lr42
LAR8p_i9_lr9
LAR8p_i44_lr21
LAR9p_i16_lr27
LAR9p_i55_lr27
LAR9p_i72_lr43
LAR14p_i13_lr32
LAR14p_i73_lr44
LAR23p_i12_lr33
LAR23p_i61_lr33
LAR23p_i74_lr45
LAR26p_i64_lr36
LAR27p_i56_lr28
LAR24c_i46_lr23
LAR26c_i47_lr24
LAR8t_i44_lr35
LAR8t_i63_lr35
LAR9c_i38_lr20
LAR8c_i37_lr19
LAR23t_i67_lr39
LAR13c_i44_lr22
LAR11c_i41_lr21
LAR11t_i76_lr47
LAR11t_i77_lr48
LAR5c_i32_lr18
Ancient humans
Tooth
Petrous
Calculus
Yersinia
relative abundance
0
42%
Modern humans
Mouth
Skin
Nose
Stool
Vagina
Soil

Figure S3. DNA fragmentation profiles. (A) DNA fragmentation profiles underlying the two ancient plague genomes characterized in this study (top: DNA library LAR8t_i63_lr35; bottom: DNA library LAR11t_i76_lr47). All aligned reads were processed using mapDamage2 (Jónsson et al., 2013) considering base with quality scores superior or equal to 30. Position-wise base compositional profiles are provided with the 10 first (left, 1 to 10) and 10 last (right, N-9 to N) read positions within sequencing reads, and the 5 genomic positions preceding read starts (left, -1 to - 5) or following read ends (right, N+1 to N+5). (B) DNA fragmentation profiles underlying the two ancient human genomes characterized in this study (top: DNA library LAR8t_i63_lr35; bottom: DNA library LAR11t_i76_lr47). Related to *Figure 2*.

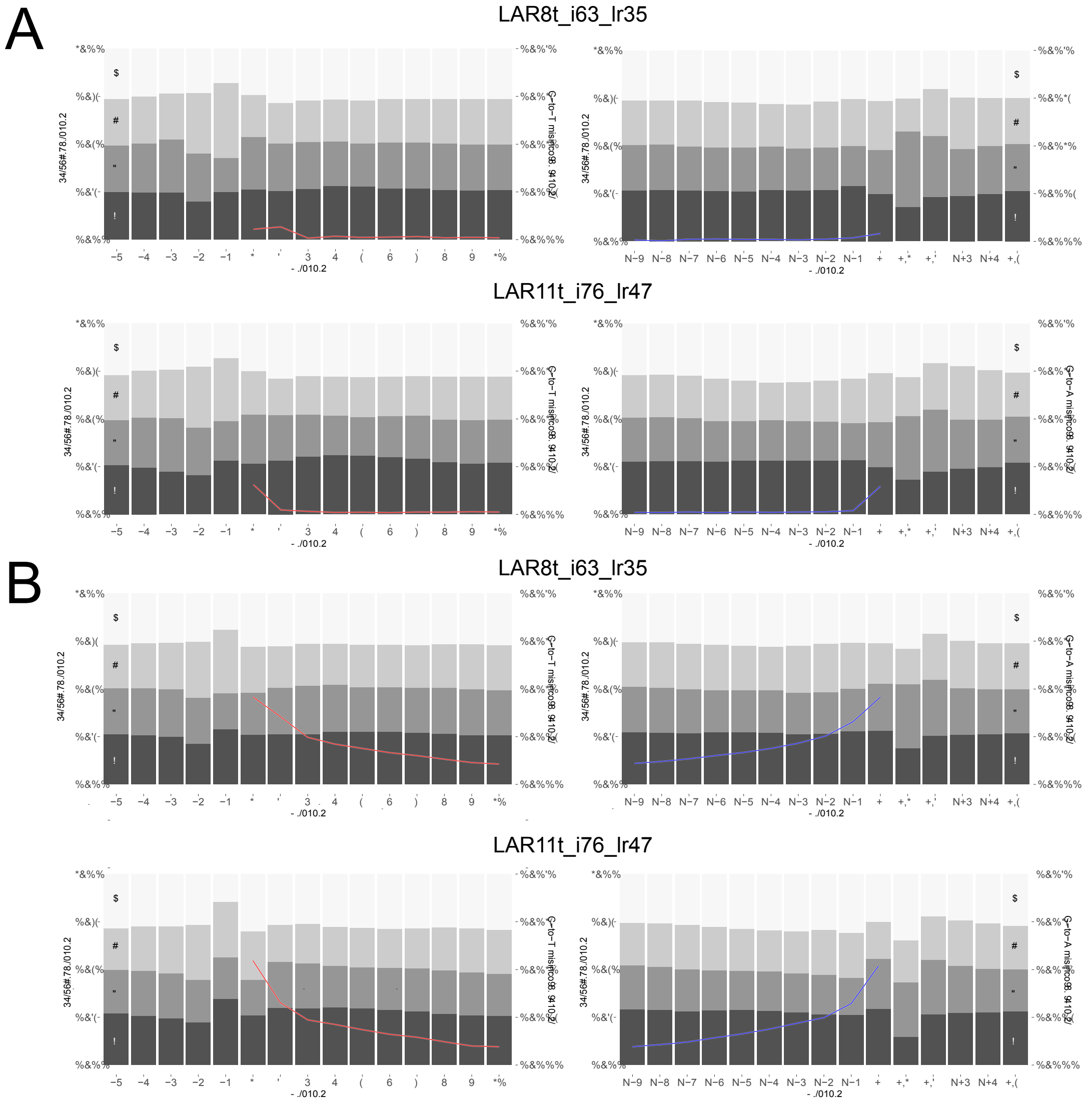

Figure S4. Nucleotide mis-incorporation profiles within CpG and CpH contexts in human and plague aligned sequencing reads. Nucleotide mis-incorporation rates are calculated within CpG and outside of CpG contexts (CpH refers to CpA, CpC and CpT contexts) in order to account for the difference in DNA methylation patterns in the human and the bacterial genome. Ancient human DNA data show evidence for higher Cytosine deamination rates than ancient bacterial DNA data in CpG contexts, in line with the presence of methylated CpGs in the human genome, that are known to undergo fast post-mortem decay (Smith et al., 2014, Seguin-Orlando et al., 2015). No differences in Cytosine mis-incorporation rates are found outside of CpG contexts across the human and bacterial data, confirming similar post-mortem deamination rates. The difference in overall Cytosine deamination rates observed by mapDamage (Figure S3) is thus fully driven by the different DNA methylation properties of plague and human genomes. (A) specimen LAR8, plague data. (B) specimen LAR11, plague data. (C) specimen LAR8, human data. (D) specimen LAR11, human data. C⟶T: Cytosine mis-incorporation rates in CpH contexts. All others: Average nucleotide mis-incorporation rates affecting adenine, guanine and thymine residues, but not cytosines. Related to Figure 2.

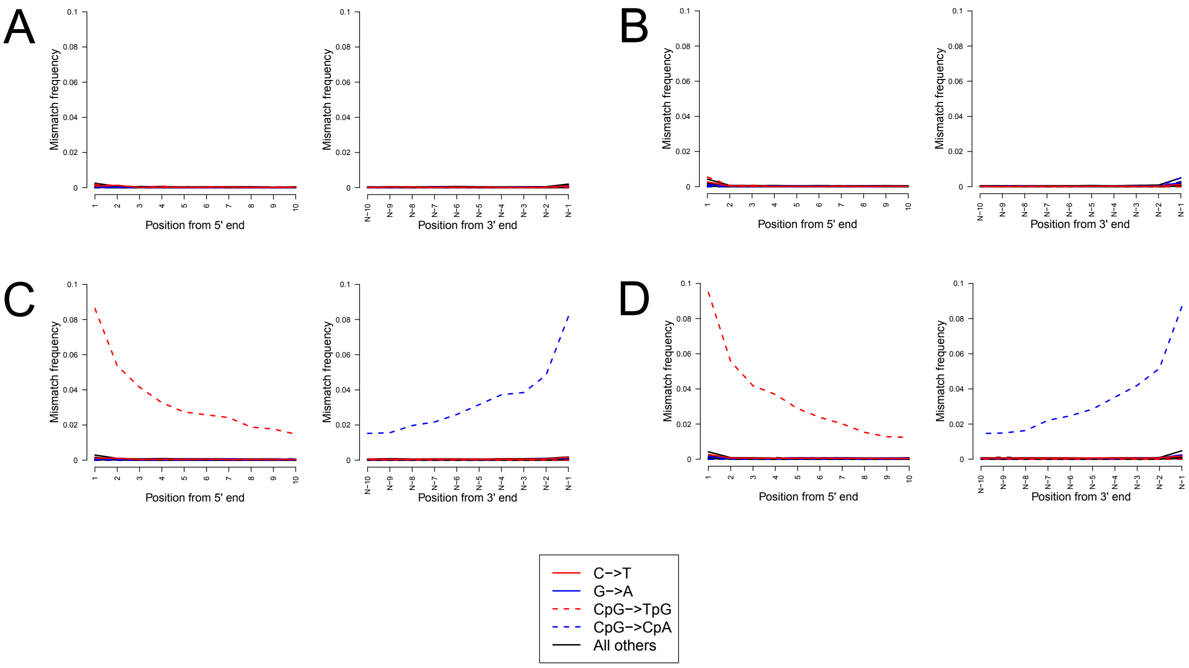

Figure S5. Human Population Genetic Affinities. (A) Principal Component Analysis (PCA) showing the two human genomes from Lariey projected onto the genetic variation present across 792 modern humans from Western Eurasia (Lazaridis et al., 2014). (B) Top-25 (Mbuti; LAR8t, X) f3-Outgroups, where X is a modern human individual from the 1240K panel. (C) Same as Panel B, for the top-25 (Mbuti; LAR11t, X) f3-Outgroups. Related to Figure 1.

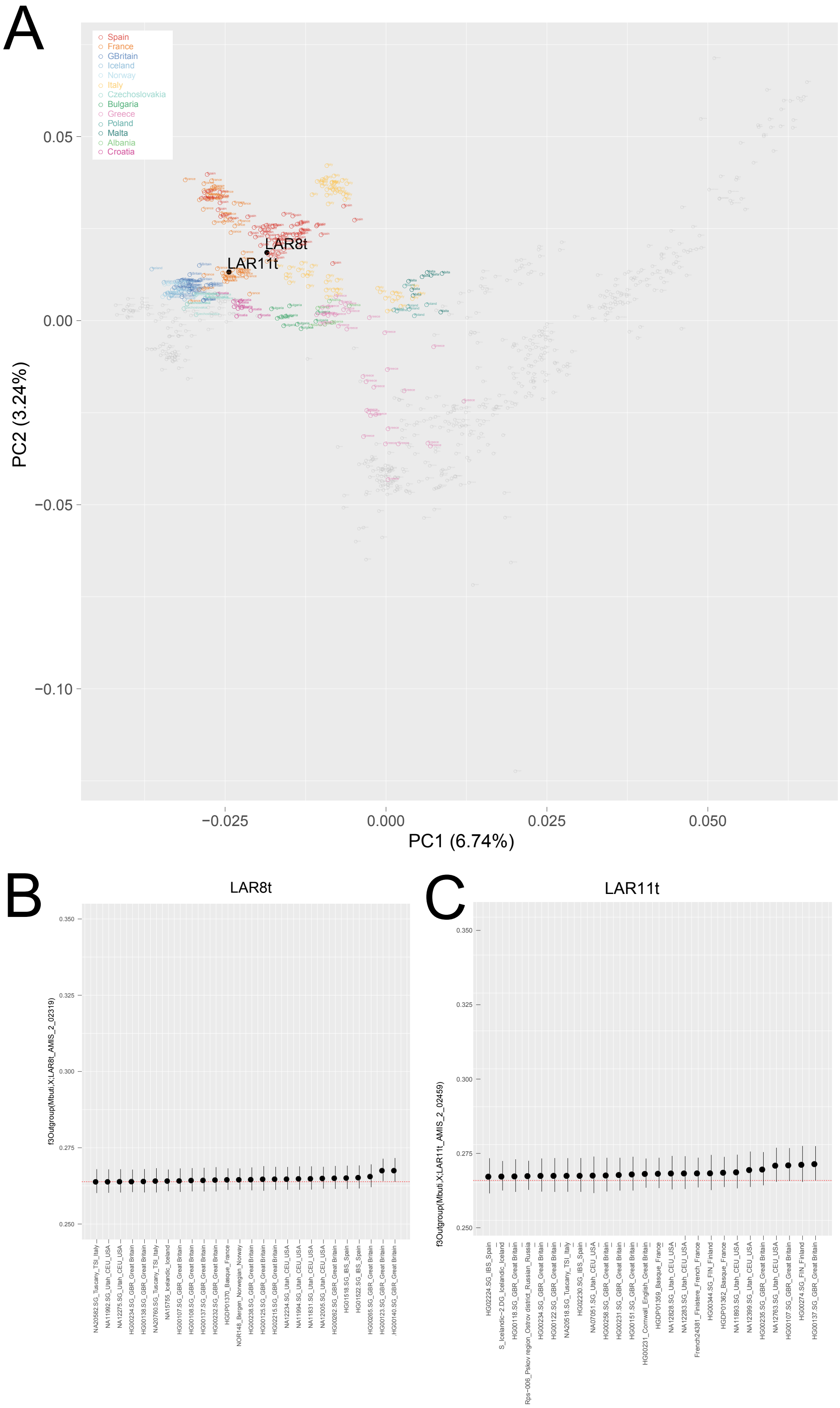
A
Spain
France
GBritain
Iceland
Norway
Italy
Czechoslovakia
Bulgaria
Greece
Poland
Malta
Albania
Croatia
LAR8t
LAR11t
0.05
0.00
−0.05
−0.10
PC2 (3.24%)
−0.025
0.000
0.025
0.050
PC1 (6.74%)
B
LAR8t
0.350
0.325
0.300
0.275
0.250
f3Outgroup(Mbuti,X;LAR8t_AMIS_2_02319)
NA20582.SG_Tuscany_TSI_Italy
NA11992.SG_Utah_CEU_USA
NA12275.SG_Utah_CEU_USA
HG00234.SG_GBR_Great Britain
HG00138.SG_GBR_Great Britain
NA20760.SG_Tuscany_TSI_Italy
NA15755_Icelandic_Iceland
HG00107.SG_GBR_Great Britain
HG00108.SG_GBR_Great Britain
HG00137.SG_GBR_Great Britain
HG00232.SG_GBR_Great Britain
HGDP01370_Basque_France
NOR148_Bergen_Norwegian_Norway
HG00238.SG_GBR_Great Britain
HG00125.SG_GBR_Great Britain
HG02215.SG_GBR_Great Britain
NA12234.SG_Utah_CEU_USA
NA11994.SG_Utah_CEU_USA
NA11831.SG_Utah_CEU_USA
NA12005.SG_Utah_CEU_USA
HG00262.SG_GBR_Great Britain
HG01518.SG_IBS_Spain
HG01522.SG_IBS_Spain
HG00265.SG_GBR_Great Britain
HG00123.SG_GBR_Great Britain
HG00140.SG_GBR_Great Britain
C
LAR11t
0.350
0.325
0.300
0.275
0.250
f3Outgroup(Mbuti,X;LAR11t_AMIS_2_02459)
HG02224.SG_IBS_Spain
S_Icelandic−2.DG_Icelandic_Iceland
HG00118.SG_GBR_Great Britain
Rps−006_Pskov region_Ostrov district_Russian_Russia
HG00234.SG_GBR_Great Britain
HG00122.SG_GBR_Great Britain
NA20518.SG_Tuscany_TSI_Italy
HG02230.SG_IBS_Spain
NA07051.SG_Utah_CEU_USA
HG00258.SG_GBR_Great Britain
HG00231.SG_GBR_Great Britain
HG00151.SG_GBR_Great Britain
HG00231_Cornwall_English_Great Britain
HGDP01359_Basque_France
NA12828.SG_Utah_CEU_USA
NA12283.SG_Utah_CEU_USA
French24381_Finistere_French_France
HG00344.SG_FIN_Finland
HGDP01362_Basque_France
NA11893.SG_Utah_CEU_USA
NA12399.SG_Utah_CEU_USA
HG00235.SG_GBR_Great Britain
NA12763.SG_Utah_CEU_USA
HG00107.SG_GBR_Great Britain
HG00274.SG_FIN_Finland
HG00137.SG_GBR_Great Britain

Figure S6. Sequence coverage profiles along the pPCP1 plasmid and the *pla* gene (red). The depth of coverage is calculated per-position and normalized to the average coverage value represented by the 90% quantile. Related to Figure 4.

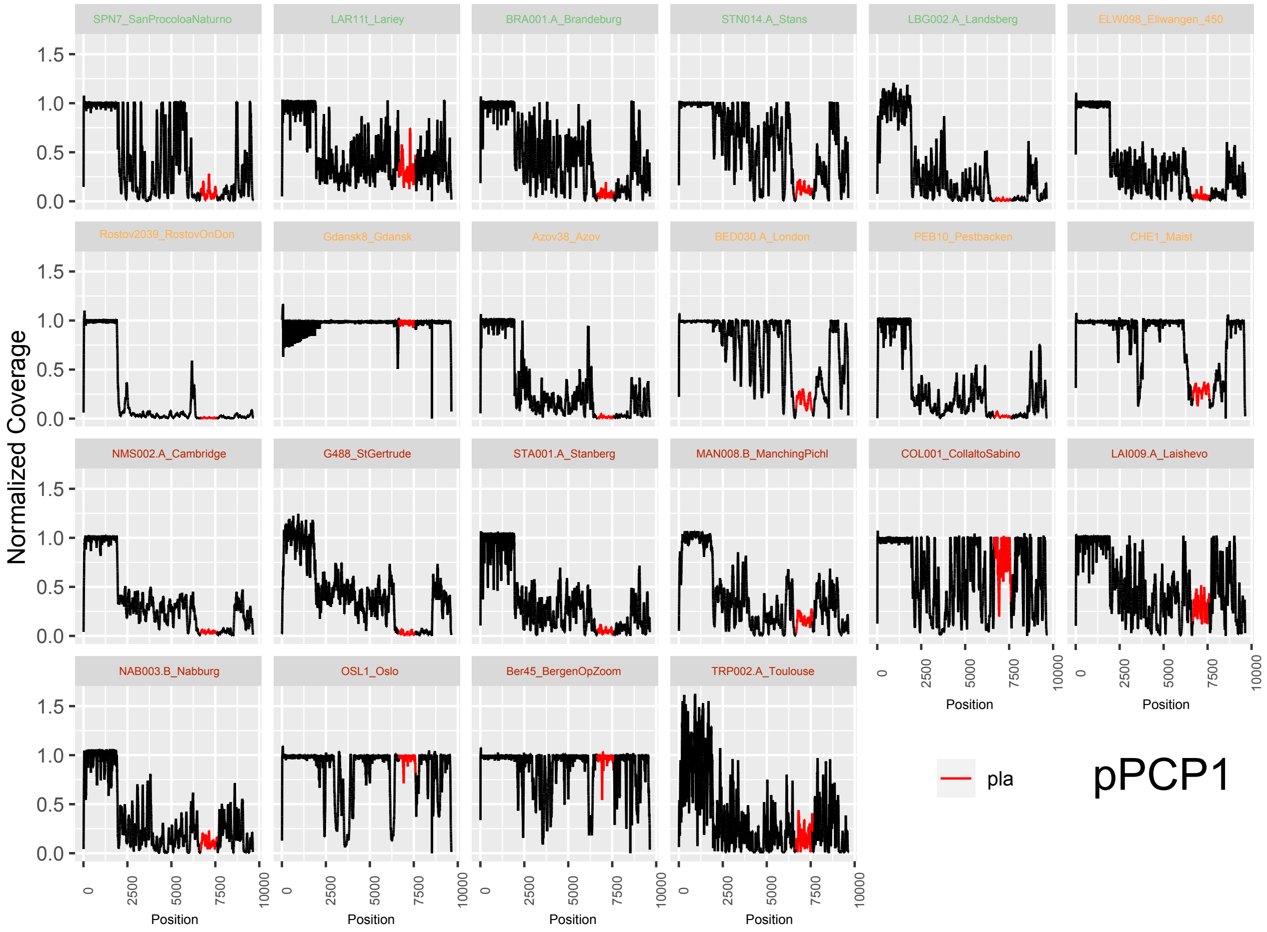

Transparent Methods

Archaeological sites, archaeological and anthropological information

The cemetery from Lariey-Puy Saint Pierre was excavated in 2002 CE (Signoli et al., 2003a) on the basis of prospective archaeological digs carried out in 2001 CE (Signoli et al., 2001). This site is located in the French Alps, approximately two kilometers west of Briançon (France), and 90 kilometers west of Turin (Italy) (Figure 1A). It represents the only French site unambiguously assigned to the 1628-1632 CE plague epidemic, based on direct radiocarbon dating of four individuals (Signoli et al., 2003a) and on the presence of rare ceramic artefacts of local origin with Piedmontese influence. In addition, historical archive confirms the presence of plague in 1630 CE in Le Pinet, one of Puy St Pierre hamlets (Briançon municipal archives). The cemetery from Lariey-Puy Saint Pierre is therefore traditionally associated with years 1629-1630 CE (Bergé, 1989; Bigny, 1982). Interestingly, the plague cemetery is still part of the local popular memory today, as testified by a wooden cross engraved "*cimetière de la peste*" (literally, standing for "*plague cemetery*" in French) erected on the site and regularly replaced, and by the annual pilgrimage organized by Puy Saint Pierre villagers for Saint-Roch's day, commemorating the Saint specially invoked against the plague. The funerary ensemble covers an area of approximately ~1,800 square meters and is delimited by a stone wall, while the cemetery *per se* is limited to ~53 square meters, in which 17 adult individuals and 17 immatures could be identified. Both individual and multiple sepultures are present on site and are displayed along a north-to-south organization (Signoli et al., 2007). While single burials are concentrated in the northern area of the funerary ensemble, the presence of double and multiple burials increases towards the south. This has suggested that the funerary ensemble was first opened from the north at the time where infection and mortality rates were still limited. As infection and mortality rates increased, bodies had to be buried together within double and multiple burials, possibly as an attempt to react promptly and prevent further spread. The LAR8 individual was identified as an adult female on the basis of the coxal morpho-anatomy (Murail et al., 2005; Brooks and Suchey, 1990; Schmitt, 2005). The LAR11 individual showed characteristic of an immature individual, probably belonging to the 15-19 age class (Schaffer et al, 2009; Scheuer and Black, 2004). Preservation states of the cranial and post-cranial skeletons of the LAR8 and LAR11 individuals are illustrated in Figure S1. Archaeological evidence indicates that LAR11 was one of the first victims of the epidemic, and was buried in a single sepulture in the most remote part of the cemetery. On the other hand, LAR8, buried in the southern part of the cemetery, was part of a group of four individuals who were buried simultaneously (LAR7, a young female adult; as well as LAR9 and LAR10: children aged between 5 and 9 years old) (Figure 1B-C; Signoli et al., 2003a). As both the administrative and scientific director of the Lariey excavation, co-author M. Signoli has obtained full official permission from the French Ministry of Culture and Communication, as well as from the regional archaeology authorities (*Service Régional de l'Archéologie* SRA PACA) to carry out sampling and scientific analyses on the material excavated (site reference 05 109 7 AH). Accordingly, the analyses presented in this study have directly involved the regional archaeology authorities SRA PACA, which are represented by co-author S. Tzortzis who took part of data collection and interpretation. The Lariey remains are currently located at the laboratory *Anthropologie bio-culturelle, droit, éthique et santé* ADES, UMR 7268, Aix-Marseille University, France. Sampling took place in February and March 2019 under the authority of co-author M. Signoli, who was then the director of this laboratory.

DNA extraction

Remains belonging to 26 different individuals were processed for this study, of which 15 were subjected to DNA sequencing and 11 were tested for the presence of *Yersinia pestis* through PCR amplification targeting a short fragment within the *pla* locus. Sample preparation was performed in the CAGT laboratory (UMR5288, Toulouse, France), following strict procedures to avoid and detect potential contamination. Samples were processed (down to the PCR setup step) in state-of-the-art ancient DNA facilities, fully dedicated to the analyses of archaeological remains and physically separated from post-PCR and modern DNA laboratories. Additionally, all experimental procedures were implemented following a systematic decontamination of material and surfaces, using disposable personal protective equipment and co-processing negative blank controls together with individual samples. For 12 individuals, DNA was extracted

from 50-200mg of petrosal bone that were obtained through the pulverization on a Retsch MM 200 instrument of a short bone piece cut with a Dremel instrument. For 23 individuals, 15-50mg of dental pulp and dentin powder was drilled from the inner surface of the tooth roots and crowns, following the procedure described by Neumann and colleagues (2020). A total of 7 teeth also showed the presence of dental calculus that we collected for DNA extraction (the weight of each sample was below the scale range, *i.e.* inferior to 10mg). For each sample (including petrosal bone, tooth and dental calculus), DNA content was extracted following the protocol described in (Seguin-Orlando et al., 2021), using 963µL of lysis buffer (0.45M EDTA, 0.25mg/mL proteinase K and 0.5% N-lauryl Sarcosyl) for a first pre-digestion step of 1 hour at 37°C, before carrying out a full digestion of the pellet overnight at 42°C in 963µL of fresh lysis buffer. Dental calculus was processed using the same extraction procedure, except that the pre-digestion step was skipped. The overnight digestion buffer was centrifuged at 12,000 rpm for 2 minutes in order to remove potentially remaining pellets, before an aliquot of 200µL of the supernatant was purified on a MinElute column (QIAGEN©) and eluted in 26µL of elution buffer (EB + 0.05% Tween 20).

PCR screening

The presence of *Yersinia pestis* DNA in the tooth extracts was assessed through a PCR assay targeting a sequence of the pPCP1 *pla* gene, as previously described (Seifert et al., 2013). A total PCR reaction volume of 25µL containing 2µL of DNA extract, 1.5 units of AmpliTaq Gold™ polymerase (ThermoFisher Scientific), 1X Gold Buffer and 200nM of each primer, was subjected to a touch-down amplification program (2 cycles of 30 seconds at 94°C, 30 seconds at 64°C and 60 seconds at 72°C, followed by 2 cycles of 30 seconds at 94°C, 30 seconds at 62°C and 60 seconds at 72°C, and finally; 46 cycles of 30 seconds at 94°C, 30 seconds at 60°C and 60 seconds at 72°C). The presence of a specific 133bp amplified product was visualized by electrophoresis on a 2% agarose gel.

Uracil excision, library construction and amplification

For a total of 29 remains, originating from 15 distinct individuals (Table S1), a 22.8µL aliquot of DNA extract was subjected to Uracil-Specific Excision Reagent treatment by incubation at 37°C for 3 hours with 7µL of USER enzyme (NEB®). Illumina sequencing libraries were constructed following a well-established protocol (originally described in (Rohland et al., 2015) and modified as described in (Fages et al., 2019)) to introduce a unique 7-nucleotide barcode within both adapters P5 and P7 (those indices are referred to hereafter as '*internal*' indices as they form the first 7 nucleotide positions within each sequencing read; see IrXX, where XX is identified as a number on Table S1). Libraries were amplified and indexed by performing 8-12 PCR cycles in 25µL reaction volumes using 1 unit of AccuPrime™ Pfx DNA polymerase, 4-6µL of DNA library and with an overall concentration of 200nM of both the InPE1.0 primer and one custom PCR primer including a unique 6-nucleotide index (this index is hereafter referred to as '*external*' index as its sequence is obtained following the priming of a sequencing reaction independent from that leading to each read pair; see iXX, where XX is identified as a number on Table S1). Amplified products were purified using Agencourt Ampure XP beads (1.4:1 or 1.6:1 as beads:DNA ratio) and eluted in 20µL EB+0.05% tween. Library molarity, size and concentration were checked on a Tapestation 4200 instrument (Agilent Technologies) and on a QuBit HS dsDNA assay (Invitrogen). Up to 3 amplified libraries were obtained per sample, for a total of 45 DNA libraries (Table S1).

DNA Sequencing

Amplified libraries were pooled with other indexed libraries and sequenced using the Paired-End mode on an Illumina MiniSeq instrument (2x80 bp reads) at the CAGT laboratory (Toulouse, France) or on a NovaSeq S4 (2x150 bp reads) at SciLifeLab (Stockholm, Sweden) in order to obtain deeper genome coverage.

Read processing, alignment, trimming and rescaling

Illumina paired-end reads were demultiplexed according to the 7-bp 'internal' indexes present at both read starts (Rohland et al., 2015), using a maximum edit distance of 1 mismatch per individual index. Demultiplexed reads were then trimmed for adapter sequences (--mm 5) and poor-quality ends (Phred quality scores ≤ 2), and collapsed into single reads using AdapterRemoval2 (Schubert et al., 2016). Collapsed, collapsed truncated and non-collapsed pairs were mapped against a number of reference genome sequences, including the human reference genome (GRCh37, hg19), the human revised Cambridge reference sequence (rCRS, Genbank Accession Number NC_120920.1), the plague reference genome (strain CO92, Accession Number = NC_003143.1 ; (Parkhill et al., 2001)), and each individual plasmid, including pCD1 (Accession Number = NC_003131.1), pMT1 (Accession Number = NC_003134.1), and pPCP1 (Accession Number = AL109969.1). As the plague chromosome is circular, the first and last 30 bp of the CO92 chromosome sequence were duplicated at the end and the start of the CO92 chromosome sequence in order to identify those reads extending across both regions. The same was done to handle the circularity of each plasmid, except that only the first 30 bp were copied at the end of each individual plasmid reference sequence. Read alignment against the human nuclear and mitochondrial genomes was carried out using Bowtie2 (Langmead et al., 2012) according to the recommendations of Poullet and Orlando (2020) and the procedures described by Seguin-Orlando and colleagues (2021). Read alignment against the plague chromosome and plasmids was carried out using BWA backtrack v0.7.17-r1194-dirty and followed the stringent parameters described by Spyrou and colleagues (2019b). All aligned reads shorter than 25 bp and alignments showing mapping quality strictly inferior to 30 were disregarded. PCR duplicates were removed using MarkDuplicates from Picard Tools (version 2.18.0, http://broadinstitute.github.io/picard/) and read were locally re-aligned around indels using GATK (version 3.8.1, (McKenna et al., 2010)). Read collapsing, trimming and mapping, as well as PCR duplicate removal and local realignment were carried out using the Paleomix automated computational pipeline (version 1.2.13.2 (Schubert et al., 2014)). Sequencing statistics, including numbers of sequencing reads, endogenous DNA content, clonality, and coverage are provided in Table S1. We downloaded previously published sequence data from 30 *Yersinia pseudotuberculosis* strains, 2 *Yersinia similis*, as well as 127 modern and 133 ancient *Yersinia pestis* strains (Table S2), representing a total of 24.8 billion reads. Raw fastq reads (or read pairs) were subjected to the same processing steps as described above so as to obtain an extensive comparative genome panel. We noticed that the ancient genomes previously characterized combined a full array of archaeological sites and experimental methods (including DNA extracts treated or not treated using the USER-enzyme (Rohland et al., 2015), shotgun sequencing and capture data). They were, thus, likely affected by various levels of post-mortem DNA damage, resulting in the presence of different proportions of nucleotide mis-incorporations in our comparative panel. In order to mitigate such effects, all plague BAM alignment files were subjected to the following procedure. First, we used PMDtools (Skoglund et al., 2014) to identify those aligned reads carrying post-mortem DNA damage signatures, using the conservative score of 1. The base quality of all read positions affected by signatures of post-mortem Cytosine deamination (i.e. C-to-T and G-to-A transitions, relative to the reference) was further downscaled using mapDamage v2.7 (Jónsson et al., 2013). This effectively resulted in the elimination of all mutations potentially introduced by post-mortem DNA damage. Once rescaled, read alignments were further trimmed across the first and last 10 positions to eliminate additional sources of spurious variation potentially present. Those aligned reads that did not show any evidence of post-mortem DNA damage (PMD score threshold < 1) were trimmed the first and last 5 read positions only. Aligned reads were then merged together using samtools merge (Li et al., 2009) in order to obtain final BAM files for downstream analyses.

Human genome analyses

Individual sex was inferred on the basis of X-to-autosomal sequence coverage. Mitochondrial haplotypes were called using haplogrep (version 2.2 (Kloss-Brandstätter et al., 2011)), minimal mapping and base quality thresholds of 30 and a minimal depth filter of 5 (or 3 for those individuals showing minimal coverage; Table S1), following the procedure from Seguin-Orlando and colleagues (2021). Contamination rates based on mitochondrial data were estimated using Schmutzi (Renaud et al., 2014). The Y-chromosome haplotype carried by individual LAR11 was called using the Yleaf statistical package (Ralf et al., 2018).

Contamination rates were also estimated for males using heterozygosity measurements at polymorphic sites present on the X chromosome, following the methodology from (Rasmussen et al., 2011) and implemented in ANGSD (Korneliussen et al., 2013), excluding transition substitutions and sites covered only once or more than 200-times. Relatedness between the LAR8 and LAR11 individuals was assessed using lcMLkin (Lipatov et al., 2015), considering all autosomal positions overlapping the 1240K dataset (v42.4 available at https://reichdata.hms.harvard.edu/pub/datasets/amh_repo/curated_releases/V42/V42.4/SHARE/public.dir/v42.4.1240K_HO.tar) and random sampling one read at those sites covered multiple times. The sequence data was converted to vcf prior to running lcMLkin using VCFtools (version 0.1.17) (Danecek et al., 2011), and pruned using the SNPbam2vcf.py script for sites located at least 100 kb apart and present at minimal 5% allelic frequencies (--thin 100000 --maf 0.05). Principal Component Analysis (PCA) was carried out using the Human Origin reference panel for 592,998 autosomal genotypes in 796 modern west Eurasian individuals, as reported by Patterson and colleagues (2012) and Lazaridis and colleagues (2014). The analysis was based on pseudo-diploid genotype calls due to the limited genome coverage achieved for both the LAR8 and LAR11 individuals. PCA was carried out using smartPCA from EIGENSOFT version 7.2.1 (Patterson et al., 2006), and turning the lsqproject and shrink options on for projecting the two ancient individuals onto the two first principal components (PCs) obtained from modern reference individuals. Finally, we carried out f3-Outgroup statistics using q3Pop from Admixtools (version 5.0 (Patterson et al., 2012)) to identify those modern individuals showing highest genetic affinities with the two ancient individuals sequenced in this study. The comparative panel included a total number of 6,152 individuals from across the world, including 4 Mbuti individuals who were used as outgroup (S_Mbuti-3.DG, B_Mbuti-4.DG, S_Mbuti-2.DG, S_Mbuti-1.DG).

Metagenomic profiling

Microbial taxonomic profiles of each individual DNA library were determined using the metaBIT automated computational package (Louvel et al., 2016), disregarding collapsed truncated and uncollapsed pairs as well as collapsed reads showing high-quality (mapping quality ≥ 30) unique alignments against the human genome, and restricting assignments to non-viral and non-eukaryotic taxa present in the MetaPhlAn2 diversity database (Truong et al., 2015). Bacterial taxa supported by abundances lower than 1% at either the species level (Figure 1D) or the genus level (Figure S2) were disregarded. Unsupervised clustering of abundance profiles was carried out at the species level using the pvclust R package (https://cran.r-project.org/web/packages/pvclust/index.html) (Suzuki and Shimodaira, 2006), assessing node support through approximately-unbiased (au) tests and 10,000 bootstrap pseudo-replicates. Principal Coordinate Analyses were carried out based on Bray-Curtis distances to compare the taxonomic profiles of all individual DNA libraries sequenced in this study. The analyses were repeated to a broader panel, including a range of modern microbial profiles, including from 15 soil samples as well as 689 human-associated modern microbiota obtained from (Fierer et al., 2012; The Human Microbiome Project Consortium, 2012). Linear Discriminant Analyses were carried out in LEfSe with default parameters (Segata et al., 2011), and grouping samples in four categories corresponding to *Yersinia pestis*-positive teeth, *Yersinia pestis*-negative teeth, petrosal bones and dental calculus.

Phylogenetic analyses

Maximum Likelihood phylogenetic trees were constructed using IQ-Tree v1.6.12 (Minh et al., 2020). The best mutational model (TVM+F+R6) was automatically selected using AICc from a panel of 283 individual models corresponding to various combinations of 22 common substitution models, site frequency models (F), and rate heterogeneity across sites (G). Base frequencies were optimized by Maximum Likelihood (ML) during phylogenetic reconstruction. Sequence alignments corresponded to those CO92 chromosome positions that were polymorphic (disregarding indels) and covered in at least half of the plague chromosomes. Any strain showing less than 75% of the resulting sites was further removed from the alignment, resulting in a total of 21,279 sites, including 17,688 parsimony-informative and 3,591 singletons. Individual base positions were called using BCFtools (version 1.8-31-g9ba4024, using htslib 1.7-41-g816a220; (https://github.com/samtools/bcftools)), especially considering minimum base and mapping

Phred scores of 30 in the mpileup module, turning the --ploidy flag to 1 in the call module, and filtering those variants located within a 10 bp range of indels and those showing a genotype Phred score strictly inferior to 30. Positions showing coverage superior to the 99.5% quantile of the position-wise coverage distribution were disregarded as potentially indicative of undetected chromosomal rearrangements such as duplications. The individual threshold per strain was obtained from the Paleomix depths command with default parameter (Schubert et al., 2014). Node support was estimated using a total of 1,000 ultrafast bootstrap (UFBoot) approximation (Hoang et al., 2018) and the SH-like approximate likelihood ratio (aLRT) test with 1,000 replicates (Guindon et al., 2010).

Bayesian phylogenetic reconstructions were carried out in BEAST v2.6.3 (Bouckaert et al., 2019), following the methodology from Spyrou and colleagues (2019b). In short, the sequence alignment used in IQ-Tree was restricted to those strains that formed the second plague pandemic as well as those belonging to the same main phylogenetic cluster, which included modern third pandemic strains (see Table S2 for the final list of samples considered). The Beast2 xml file was prepared in BEAUti 2 (Drummond et al., 2012), importing the fasta alignment and providing absolute dates for tips (before present). The GTR+G8 substitution model was considered and mutation rate variation (uniform prior range between 1.e-2 to 1.e-10 substitution per site per year) along the tree branch lengths was modeled using the Relaxed Clock Log Normal clock model (Drummond et al., 2006). Root-to-top regression in TempEst v1.5.3 (http://tree.bio.ed.ac.uk/software/tempest/) using the IQtree ML-tree obtained on the data underlying the Beast2 analysis, and constructed without molecular clock assumption, supported temporal structure in the data (R squared function, correlation coefficient = 0.769). Effective population size could vary according to the stepwise coalescent Skyline model (Drummond et al., 2005), considering a total of 20 individual time bins. The tree topology, branch length and the other model parameters were estimated following 750,000,000 MCMC iterations, sampling 1 very 100 state. A final set of 50,000 trees was obtained using LogCombiner disregarding the first 20% as burn-in and sampling a final subset of 50,000 trees with LogCombiner (https://www.beast2.org/programs/). The maximum clade credibility tree was obtained in TreeAnnotator (https://www.beast2.org/treeannotator/). MCMC convergence was assessed through ESS values superior to 200 for all relevant model parameters. All trees were plotted using the R ggtree library (Yu et al., 2018).

Genome characteristics

Patterns of depth-of-coverage variation along the CO92 chromosome were calculated for each individual strain using 1,000 bp non-overlapping sliding windows using the Paleomix coverage command (Schubert et al., 2014) (Figure 2). Depth-of-overage calculations were carried out within 100 bp non-overlapping sliding windows for the three individual plasmids. Additionally, patterns of %GC variation along the CO92 chromosome and the three plasmids were calculated within the same windows and using seqtk (https://github.com/lh3/seqtk). Circular plots were plotted using the R circlize library (Gu, 2014). Gene coverage was calculated using the Paleomix depths module (Schubert et al., 2014) for a total of 163 chromosome and 44 plasmid regions previously described to be associated with virulence characteristics (Zhou et al., 2004; Zhou and Yang, 2009; Keller et al., 2019; Spyrou et al., 2019b). Here, gene coverage was calculated as the fraction of the positions that was covered at least once. Gene coverage was plotted using the heatmap function from the R ggplot2 library (https://cran.r-project.org/web/packages/ggplot2/index.html) (Figure 4A). Synonymous and non-synonymous sequence polymorphisms were annotated using snpToolkit v2.0.6 (Namouchi et al., 2018) and the individual vcf files generated as described in the previous section. The minimal depth threshold was set to 3 (DP4≥3) for bases showing Phred scores above or equal to 30. The allele present in a given strain was then identified as long as it was present in at least 90% of the reads. We also calculated the overall heterozygosity observed along the CO92 chromosome using VCFtools and the same parameters as those described in the previous section, except that the –ploidy option was set to 2. Edit distance distributions were generated from the NM:i field obtained while running the samtools view command. Coverage variation along the pPCP1 plasmid shown as Figure S6 is calculated per-position and normalized to the average coverage value represented by the 90% quantile.

Data availability

Raw sequence data and alignments are available at the European Nucleotide Archive (ENA) under accession number PRJEB43291.

Supplemental References

Bergé, F. (1989). Puy-Saint-Pierre : aperçu historique, mémoire d'un village (Escartons Briançon).

Bligny, B. (1982). Histoire du Dauphiné (Privat).

Brooks, S. and Suchey, J. (1990). Skeletal age determination base on the Os Pubis: A Comparison of the Acsádi-Nemeskéri and Suchey-Brooks Methods. Hum Evol, *5*, 227-238.

Danecek, P., Auton, A., Abecasis, G., Albers, C.A., Banks, E., DePristo, M.A., Handsaker, R.E., Lunter, G., Marth, G.T., Sherry, S.T., et al. (2011). The variant call format and VCFtools. Bioinformatics *27*, 2156–2158.

Drummond, A.J., Rambaut, A., Shapiro, B., and Pybus, O.G. (2005). Bayesian coalescent inference of past population dynamics from molecular sequences. Mol. Biol Evol. *22*, 1185–1192.

Drummond, A.J., Ho, S.Y.W., Phillips, M.J., and Rambaut, A. (2006). Relaxed Phylogenetics and Dating with Confidence. PLoS Biol. *4*, e88.

Drummond, A.J., Suchard, M.A., Xie, D., and Rambaut, A. (2012). Bayesian phylogenetics with BEAUti and the BEAST 1.7. Mol. Biol. Evol. *29*, 1969–1973.

Fages, A., Hanghøj, K., Khan, N., Gaunitz, C., Seguin-Orlando, A., Leonardi, M., McCrory Constantz, C., Gamba, C., Al-Rasheid, K.A.S., Albizuri, S., et al. (2019). Tracking Five Millennia of Horse Management with Extensive Ancient Genome Time Series. Cell *177*, 1419-1435.e31.

Fierer, N., Leff, J.W., Adams, B.J., Nielsen, U.N., Bates, S.T., Lauber, C.L., Owens, S., Gilbert, J.A., Wall, D.H., and Caporaso, J.G. (2012). Cross-biome metagenomic analyses of soil microbial communities and their functional attributes. Proc. Natl. Acad. Sci. U S A *109*, 21390–21395.

The Human Microbiome Project Consortium (2012). A framework for human microbiome research. Nature *486*, 215–221.

Korneliussen, T.S., Moltke, I., Albrechtsen, A., and Nielsen, R. (2013). Calculation of Tajima's D and other neutrality test statistics from low depth next-generation sequencing data. BMC Bioinformatics *14*, 289.

Kloss-Brandstätter, A., Pacher, D., Schönherr, S., Weissensteiner, H., Binna, R., Specht, G., Kronenberg, F. (2011). HaploGrep: a fast and reliable algorithm for automatic classification of mitochondrial DNA haplogroups. Hum. Mutat. *32*, 25–32.

Langmead, B., and Salzberg, S.L. (2012). Fast gapped-read alignment with Bowtie 2. Nat. Methods 9, 357–359.

Lazaridis, I., Patterson, N., Mittnik, A., Renaud, G., Mallick, S., Kirsanow, K., Sudmant, P.H., Schraiber, J.G., Castellano, S., Lipson, M., et al. (2014). Ancient human genomes suggest three ancestral populations for present-day Europeans. Nature *513*, 409–413.

Li, H., Handsaker, B., Wysoker, A., Fennell, T., Ruan, J., Homer, N., Marth, G., Abecasis, G., Durbin, R., 1000 Genome Project Data Processing Subgroup. (2009). The Sequence Alignment/Map format and SAMtools. Bioinformatics *25*, 2078–2079.

Lipatov, M., Sanjeev, K., Patro, R. and Veeramah, K. (2015). Maximum Likelihood Estimation of Biological Relatedness from Low Coverage Sequencing Data. doi:10.1101/023374.

McKenna, A., Hanna, M., Banks, E., Sivachenko, A., Cibulskis, K., Kernytsky, A., Garimella, K., Altshuler, D., Gabriel, S., Daly, M., et al. (2010). The Genome Analysis Toolkit: A MapReduce framework for analyzing next-generation DNA sequencing data. Genome Res. *20*, 1297–1303.

Murail, P., Bruzek, J., Houët, F. and Cunha, E. (2005). DSP : a tool for probabilistic sex diagnosis using worlwide variability in hip bone measurements. BMSAP *17*, 167-176.

Neumann, G.U., Andrades Valtueña, A., Fellows Yates, J.A., Stahl, R., and Brandt, G. (2020). Tooth Sampling from the inner pulp chamber for ancient DNA Extraction v1 (protocols.io.bakqicvw). https://doi.org/10.17504/protocols.io.bakqicvw

Patterson, N., Moorjani, P., Luo, Y., Mallick, S., Rohland, N., Zhan, Y., Genschoreck, T., Webster, T., and Reich, D. (2012). Ancient admixture in human history. Genetics *192*, 1065–1093.

Poullet, M., and Orlando, L. (2020). Assessing DNA Sequence Alignment Methods for Characterizing Ancient Genomes and Methylomes. Frontiers Ecol. Evol. *8*, 105.

Ralf, A., Montiel González, D., Zhong, K., and Kayser, M. (2018). Yleaf: Software for Human Y-Chromosomal Haplogroup Inference from Next-Generation Sequencing Data. Mol. Biol. Evol. *35*, 1291–1294.

Rasmussen, M., Guo, X., Wang, Y., Lohmueller, K.E., Rasmussen, S., Albrechtsen, A., Skotte, L., Lindgreen, S., Metspalu, M., Jombart, T., et al. (2011). An Aboriginal Australian genome reveals separate human dispersals into Asia. Science *334*, 94–98.

Renaud, G., Slon, V., Duggan, A.T. and Kelso, J. (2015). Schmutzi: estimation of contamination and endogenous mitochondrial consensus calling for ancient DNA. Genome Biol. *16*, 224.

Schaefer, M., Black, S. and Scheuer, L. (2009) Juvenile Osteology: A laboratory and Field Manual (Academic Press London).

Scheuer, L. and Black, S. (2004).The juvenile skeleton. (Academic Press London).

Schmitt, A. (2005). Une nouvelle méthode pour estimer l'âge au décès des adultes à partir de la surface sacro-pelvienne iliaque. *BMSAP 17, 1-13.*

Schubert, M., Lindgreen, S. and Orlando, L. (2016). AdapterRemoval v2: rapid adapter trimming, identification, and read merging. BMC Res Notes *9*, 88.

Seguin-Orlando, A., Donat, R., Der Sarkissian, C., Southon, J., Thèves, C., Manen, C., Tchérémissinoff, Y., Crubézy, E., Shapiro, B., Deleuze, J.F., et al. (2021). Heterogeneous hunter-gatherer and steppe-related ancestries in Late Neolithic and Bell Beaker genomes from present-day France. Curr. Biol. https://doi.org/10.1016/j.cub.2020.12.015.

Seifert, L., Harbeck, M., Thomas, A., Hoke, N., Zöller, L., Wiechmann, I., Grupe, G., Scholz, H.C. and Riehm, J.M. (2013). Strategy for Sensitive and Specific Detection of *Yersinia pestis* in Skeletons of the Black Death Pandemic. PLoS ONE *8*, e75742.

Signoli, M. (2001). *Le cimetière des pestiférés de Lariey, (Puy-Saint-Pierre, Hautes-Alpes), rapport d'opération de sondages* (SRA PACA).

Signoli, M., Tzortzis, S., Bizot, B., Ardagna, Y., Rigeade, C., and Seguy, I. (2007). Découverte d'un cimetière de pestiférés du XVIIème siècle (Puy-Saint-Pierre, Hautes-Alpes, France). *In* LA PESTE : entre épidémies et sociétés*, Signoli, M., Cheve, D., Adalian, P., Boëtsch, G. and Dutour, O., ed. (*Firenze University Press).

Skoglund, P., Northoff, B.H., Shunkov, M.V., Derevianko, A.P., Pääbo, S., Krause, J., and Jakobsson, M. (2014). Separating endogenous ancient DNA from modern day contamination in a Siberian Neandertal. Proc. Natl. Acad. Sci. U S A *111*, 2229–2234.

Yu, G., Lam, T.T.-Y., Zhu, H., and Guan, Y. (2018). Two methods for mapping and visualizing associated data on phylogeny using ggtree. Mol. Biol. Evol *35*, 3041–3043.

Zhou, D., Tong, Z., Song, Y., Han, Y., Pei, D., Pang, X., Zhai, J., Li, M., Cui, B., Qi, Z., et al. (2004). Genetics of Metabolic Variations between *Yersinia pestis* Biovars and the Proposal of a New Biovar, *microtus*. J. Bacteriol. *186*, 5147–5152.

Zhou, D., and Yang, R. (2009). Molecular Darwinian Evolution of Virulence in *Yersinia pestis.* Infect. Immun. *77*, 2242–2250.
